# Supplementary material for: Spatially patterned hydrogen peroxide orchestrates stomatal development in Arabidopsis
Source: Nat Commun. 2022 Aug 26;13:5040. doi: 10.1038/s41467-022-32770-7 (PMC9418256; doi:10.1038/s41467-022-32770-7)
Supplement: Supplementary file 1 — Supplementary Information [file 41467_2022_32770_MOESM1_ESM.pdf]

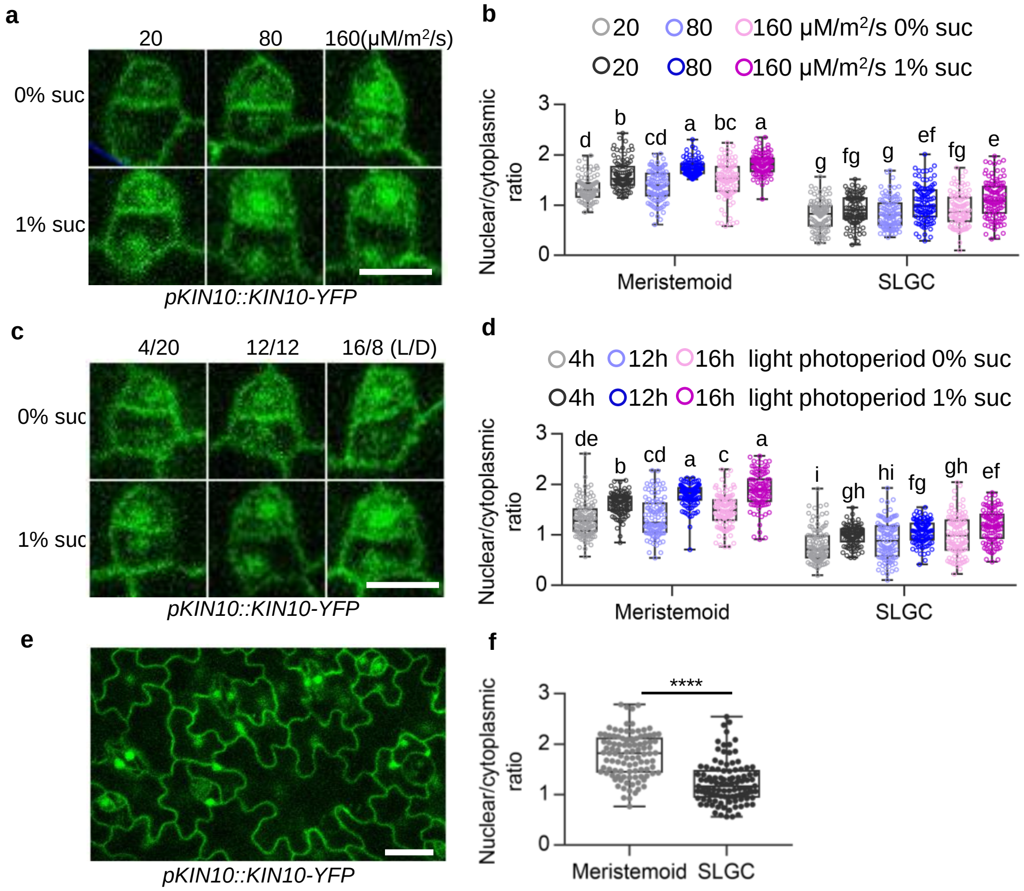

Supplementary Fig. 1 The enriched nuclear-localization pattern of KIN10 in meristemoid cells is widespread in the plants grown under different conditions.

a-b, Confocal microscopic analysis of subcellular localization of KIN10 in the cotyledon epidermal cells of plants grown under different light intensity conditions. *n* = 105, *n* = 106, *n* = 104, *n* = 104, *n* = 102, and *n* = 114 meristemoid or SLGC cells in 10 cotyledons were analyzed by ImageJ software in (**b**). Seedlings of *pKIN10::KIN10-YFP* were grown on ½ solid MS medium with or without 1% sucrose under 16h light/8h dark photoperiod with different light intensity for 4 days. **c-d**, Confocal microscopic analysis of subcellular localization of KIN10 in the cotyledon epidermal cells of plants grown under different photoperiod conditions. *n* = 117, *n* = 103, *n* = 108, *n* = 104, *n* = 108, and *n* = 104 meristemoid or SLGC cells in 10 cotyledons were analyzed by ImageJ software in (**d**). Seedlings of *pKIN10::KIN10-YFP* were grown on ½ MS solid medium with or without 1% sucrose under different photoperiod with 100 μMol/m^2/s for 4 days. **e-f**, The subcellular localization of KIN10-YFP in the true leaves. *n* = 103 meristemoid or SLGC cells in 10 true leaves were analyzed by ImageJ software in (**f**). Seedlings of *pKIN10::KIN10-YFP* were grown on ½ MS solid medium containing 1% sucrose under 16h light/8h dark photoperiod with 100 μMol/m^2/s for 12 days.

Serial Z-stack projection images were used for quantitative analysis. Scale bars in confocal images represent 10 μm in (**a**, **c**) and in (**e**) represent 20 μm. Box plot shows maxima, first quartile, median, third quartile, minima. Different letters above the bars indicated statistically significant differences between the samples (Brown-Forsythe ANOVA analysis followed by Dunnett's T3 multiple comparisons test, $p < 0.05$). Adjustments were made for multiple comparisons test). Asterisk between the bars indicated statistically significant differences between the samples (Two-tailed student's t test, ****$p < 0.0001$).

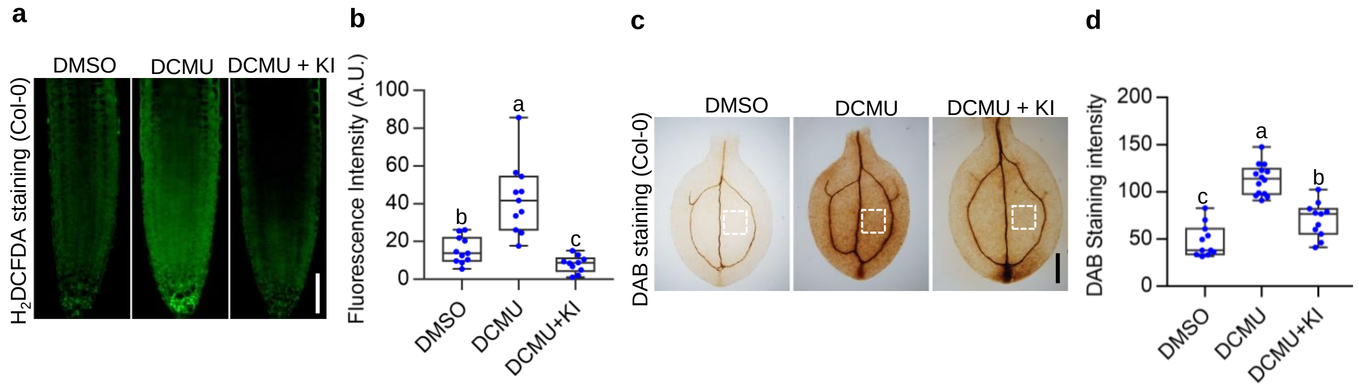

Supplementary Fig. 2 KI counteracts the DCMU effects on H_2O_2 accumulation.

a-b, H_2DCFDA staining in the primary root tips of Col-0 plants treated with or without DCMU and KI. n = 11 (DMSO), n = 11 (DCMU) and n = 10 (DCMU + KI) independent cotyledons were examined in (**b**). **c-d**, DAB staining in the leaves of Col-0 plants treated with or without DCMU and KI. n = 11 (DMSO), n = 14 (DCMU) and n = 11 (DCMU + KI) independent cotyledons were examined in (**d**). The mid leaf region of the cotyledon was used for analyzing the H_2O_2 content by Scion Image software.

Seedlings of Col-0 were grown on ½ MS solid medium containing 1% sucrose under 16h light/8h dark photoperiod with 100 μMol/m^2/s for 5 days, and then transferred to ½ MS solid medium with or without 50 μM DCMU and/or 1 mM KI for 12 hours. Fluorescent signals were taken using LSM700 microscope from Zeiss. Scale bars in panel (**a**) represent 20 μm, and in panel (**c**) represent 200 μm. Box plot shows maxima, first quartile, median, third quartile, minima. Different letters above the bars indicated statistically significant differences between the samples (Brown-Forsythe ANOVA analysis followed by Dunnett's T3 multiple comparisons test, $p < 0.05$ (**b**); One-way ANOVA analysis followed by Tukey's multiple comparisons test, $p < 0.05$ (**d**)). Adjustments were made for multiple comparisons test.

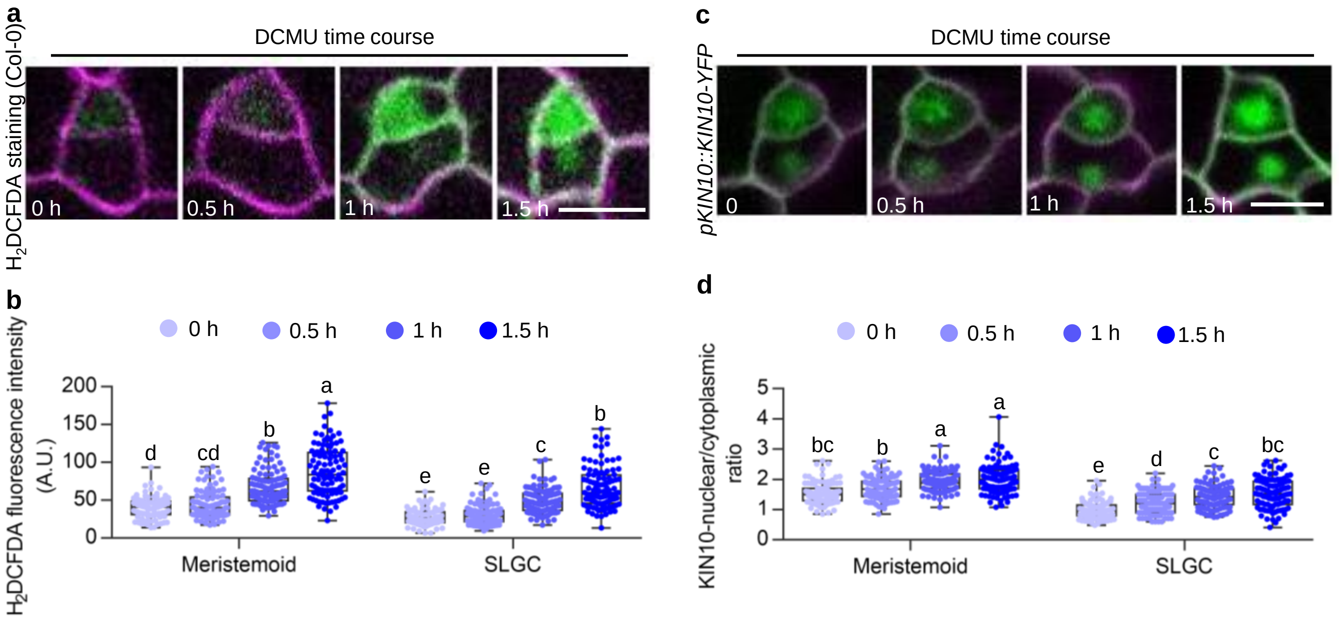

Supplementary Fig. 3 DCMU promotes the H_2O_2 accumulation and induces the nuclear localization of KIN10.

a-b, H_2DCFDA staining in the epidermal cells of leaves in response to DCMU. n = 102 (0 h), n = 101 (0.5 h), n = 103 (1 h) and n = 105 (1.5 h) meristemoid or SLGC cells in 10 cotyledons were analyzed by ImageJ software in (**b**). **c-d**, DCMU induced the nuclear localization of KIN10. n = 102 (0 h), n = 101 (0.5 h), n = 103 (1 h) and n = 105 (1.5 h) meristemoid or SLGC cells in 10 cotyledons were analyzed by ImageJ software in (**d**).

Seedlings of wild type Col-0 and *pKIN10::KIN10-YFP* transgenic plants were grown on ½ MS solid medium containing 1% sucrose under 16h light/8h dark photoperiod with 100 μMol/m^2/s for 4 days, and then sprayed with or without 50 μM DCMU for different times. Fluorescent signals were taken using LSM700 microscope from Zeiss. Scale bars in confocal images represent 10 μm. Box plot shows maxima, first quartile, median, third quartile, minima. Different letters above the bars indicated statistically significant differences between the samples (Brown-Forsythe ANOVA analysis followed by Dunnett's T3 multiple comparisons test, $p < 0.05$). Adjustments were made for multiple comparisons test.

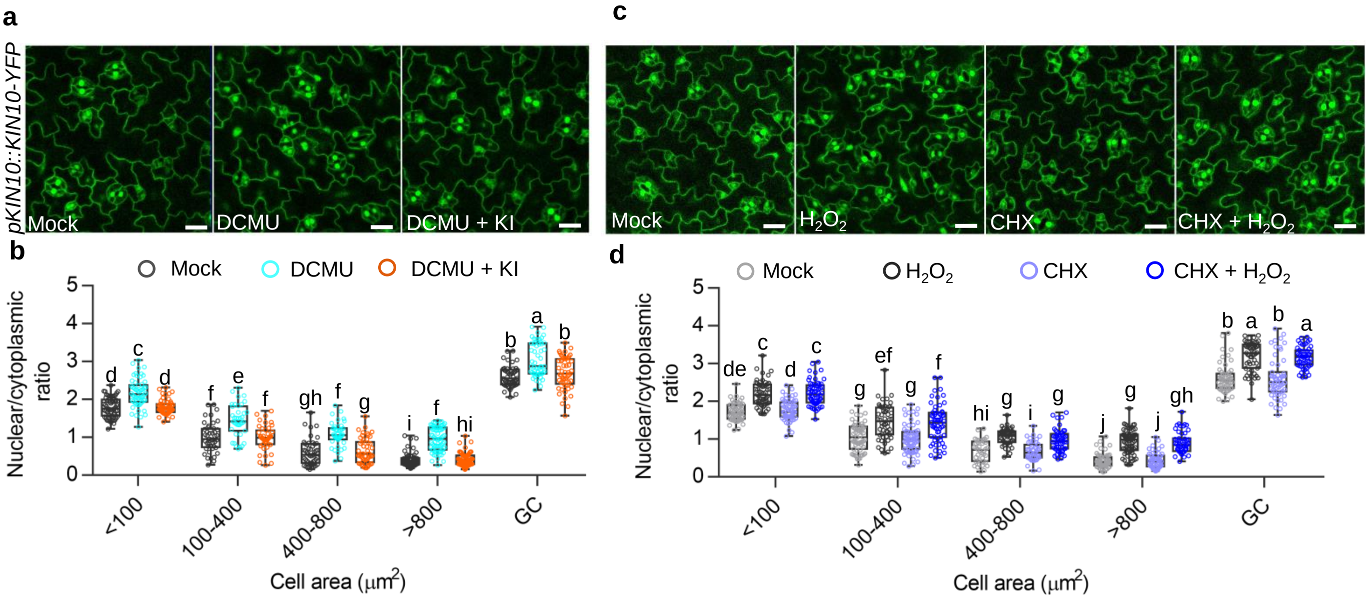

Supplementary Fig. 4 H_2O_2 induces the nuclear localization of KIN10.

a-b, KI prevented DCMU-induced nuclear localization of KIN10. **c-d**, CHX had no significant effects on H_2O_2-induced nuclear localization of KIN10 in plants.

Seedlings of *pKIN10::KIN10-YFP* were grown on ½ MS solid medium containing 1% sucrose under 16h light/8h dark photoperiod with 100 µMol/m^2/s for 4 days, and then treated with or without 50 µM DCMU and/or 1 mM KI for 12 hours (**a**, **b**) or treated with or without 2 mM H_2O_2 and/or 50 µM CHX for 2 hours (**c**, **d**). Nuclear and cytoplasmic KIN10-YFP signal from more than 200 epidermal cells in 10 cotyledons were analyzed by ImageJ software. Serial Z-stack projection images were used for quantitative analysis. Scale bars in confocal images represent 20 µm. Box plot shows maxima, first quartile, median, third quartile, minima. Different letters above the bars indicated statistically significant differences between the samples (Brown-Forsythe ANOVA analysis followed by Dunnett's T3 multiple comparisons test, $p < 0.05$). Adjustments were made for multiple comparisons test.

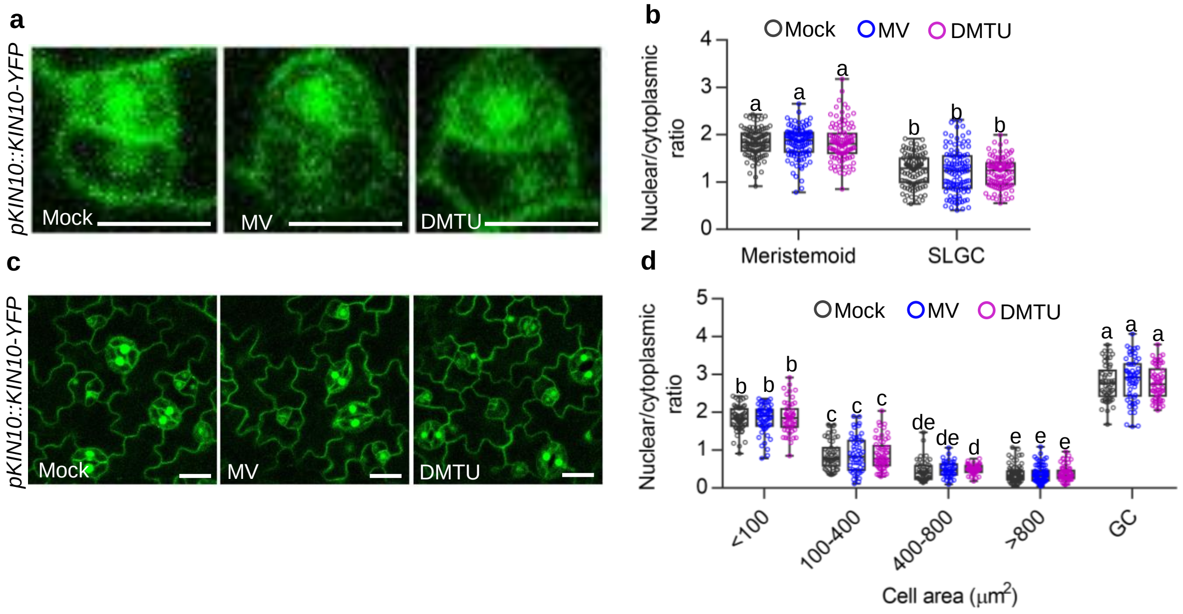

Supplementary Fig. 5 MV and DMTU have no significant effects on the nuclear localization of KIN10-YFP.

a-d, Quantification of MV and DMTU effects on the nuclear localization of KIN10-YFP. *n* = 106 (Mock), *n* = 113 (MV), and *n* = 105 (DMTU) meristemoid or SLGC cells in 10 cotyledons were analyzed by ImageJ software in (**b**). Seedlings of *pKIN10::KIN10-YFP* were grown on ½ MS solid medium with 1% sucrose under 16h light/8h dark photoperiod with 100 µMol/m^2/s for 4 days, and then treated with or without 1 µM MV or 10 mM DMTU for 6 hours. Nuclear and cytoplasmic KIN10-YFP signal from more than 200 epidermal cells in 10 cotyledons were analyzed by ImageJ software. Serial Z-stack projection images were used for quantitative analysis. Scale bars in confocal images represent 10 µm in (**a**) and 20 µm in (**c**). Box plot shows maxima, first quartile, median, third quartile, minima. Different letters above the bars indicated statistically significant differences between the samples (Brown-Forsythe ANOVA analysis followed by Dunnett's T3 multiple comparisons test, $p < 0.05$). Adjustments were made for multiple comparisons test.

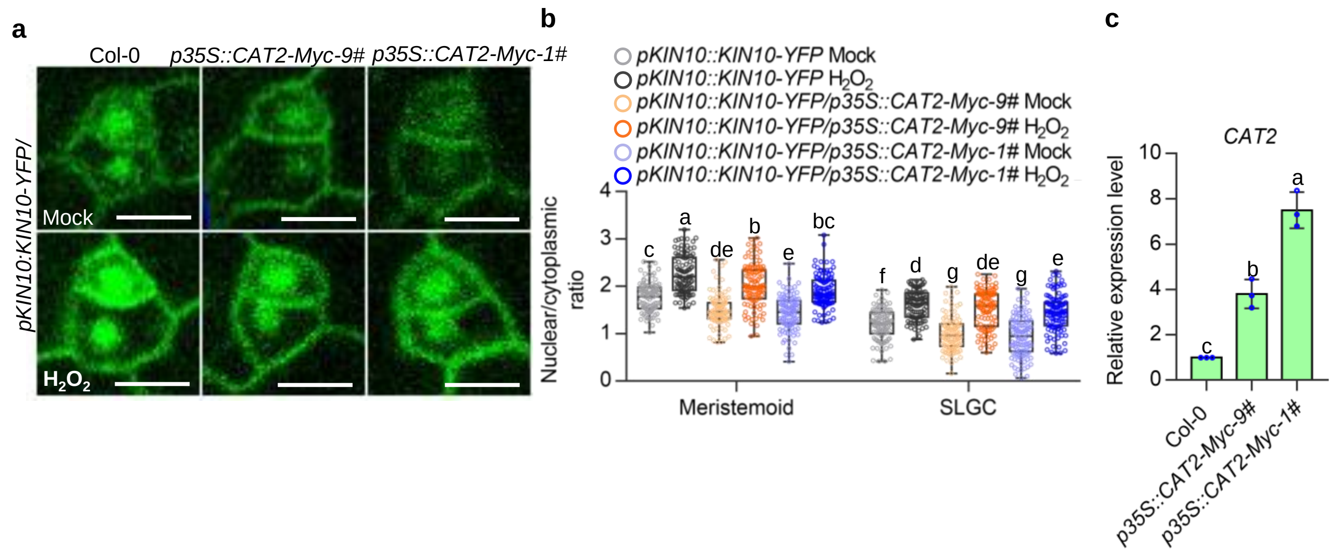

Supplementary Fig. 6 Overexpression of *CAT2* reduces the nuclear localization of KIN10 in plants.

a-b, The subcellular localization of KIN10-YFP in wild type or *p35S::CAT2-Myc* plants. Seedlings of *pKIN10::KIN10-YFP* and *pKIN10:KIN10-YFP/p35S::CAT2-Myc* were grown on ½ MS solid medium containing 1% sucrose under 16h light/8h dark photoperiod with 100 μMol/m^2/s for 4 days, and then treated with or without 2 mM H_2O_2 for 3 hours. Serial Z-stack projection images were used for quantitative analysis. n = 101 (Col-0-Mock), n=105 (Col-0-H_2O_2), n = 101 (*p35S::CAT2-9#*-Mock), n = 103 (*p35S::CAT2-9#*-H_2O_2), and n = 108 (*p35S::CAT2-1#*-Mock), n = 111 (*p35S::CAT2-1#*-H_2O_2) meristemoid or SLGC cells in 10 cotyledons were analyzed by ImageJ software in (**b**). Scale bars in confocal images represent 10 μm. Box plot shows maxima, first quartile, median, third quartile, minima. Different letters above the bars indicate statistically significant differences between the samples (Brown-Forsythe ANOVA analysis followed by Dunnett's T3 multiple comparisons test, $p < 0.05$. Adjustments were made for multiple comparisons test). **c**, Quantitative RT-qPCR analysis of the expression of *CAT2* in wild type and *p35S::CAT2-Myc* transgenic plants. Seedlings of Col-0 and two *p35S::CAT2-Myc* transgenic lines were grown on ½ MS solid medium containing 1% sucrose under 16h light/8h dark photoperiod with 100 μMol/m^2/s for 5 days. *PP2A* gene was used as an internal control. Error bars indicate standard deviation (S.D.) (n=3 biologically independent samples). Different letters above the bars indicate statistically significant differences between the samples (One-way ANOVA analysis followed by Tukey's multiple comparisons test, $p < 0.05$. Adjustments were made for multiple comparisons test).

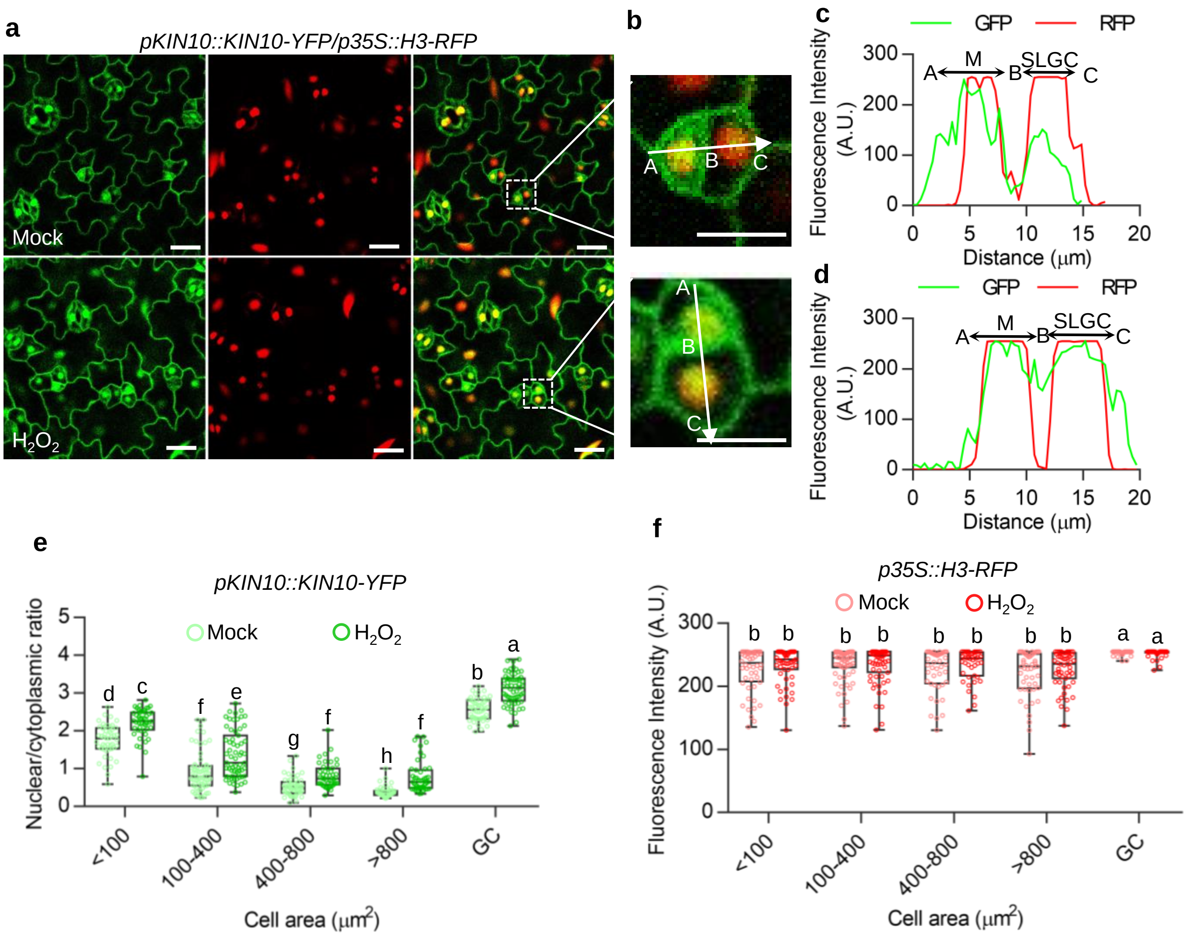

Supplementary Fig. 7 Quantification of H_2O_2 effects on the subcellular localization of KIN10-YFP and Histone3-RFP in the *pKIN10::KIN10-YFP/p35S::H3-RFP* transgenic plants.

a-d, The subcellular localization of KIN10-YFP and Histone3-RFP in response to H_2O_2. Seedlings of *pKIN10::KIN10-YFP/p35S::H3-RFP* were grown on ½ MS solid medium containing 1% sucrose under 16h light/8h dark photoperiod with 100 μMol/m^2/s for 4 days, and then treated with or without 2 mM H_2O_2 for 3 hours. Magnifications of the epidermal cells are shown in (**b**). The fluorescent signals of GFP and RFP were determined along a line drawn on the confocal images using ImageJ software. The arrow labeling with A and B represents the fluorescent signals in meristemoid cells, and the arrow labeling with B and C represents the fluorescent signals in SLGC. **e**, Quantification of nuclear localization of KIN10 in different scale epidermal cells. **f**, Quantification of nuclear localization of Histone 3 in different scale epidermal cells. Signals were taken using LSM700 microscope from Zeiss. Nuclear and cytoplasmic KIN10-YFP and Histone3-RFP signal from more than 200 epidermal cells in 10 cotyledons were analyzed by ImageJ software in (**e**), (**f**) respectively. Scale bars in panel (**a**) represent 20 μm, and in panel (**b**) represent 10 μm. Box plot shows maxima, first quartile, median, third quartile, minima. Different letters above the bars indicated statistically significant differences between the samples (Brown-Forsythe ANOVA analysis followed by Dunnett's T3 multiple comparisons test, $p < 0.05$. Adjustments were made for multiple comparisons test.).

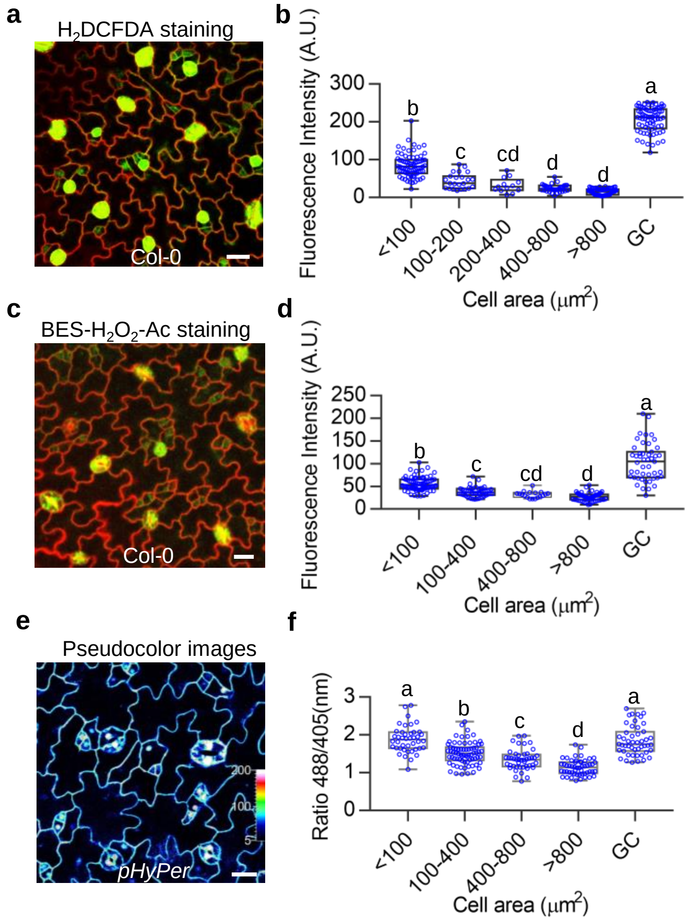

Supplementary Fig. 8 The spatial pattern of H_2O_2 in epidermal leaves.

a-b, Quantification of H_2DCFDA fluorescent intensities in the epidermal cells of cotyledon leaves. **c-d**, Quantification of BES-H_2O_2-Ac fluorescent intensities in the epidermal cells of cotyledon leaves. **e-f**, Quantification of *pHyPer* fluorescent signals in cotyledon epidermal cells. Ratio imaging of Arabidopsis epidermal cells expressing *HyPer*. Seedlings of wild-type plants or *pHyPer* transgenic plants were grown on ½ MS solid medium containing 1% sucrose under 16h light/8h dark photoperiod with 100 μMol/m^2/s for 4 days. Fluorescent signals were taken using LSM700 microscope from Zeiss. The fluorescent signal from more than 200 epidermal cells in 10 cotyledons were analyzed by ImageJ software in (**b**), (**d**), (**f**) respectively. Box plot showed the negative relationship of the fluorescent intensities of H_2DCFDA, BES-H_2O_2-Ac or *pHyPer* and the sizes of epidermal cells. Scale bars in confocal images represent 20 μm. Box plot shows maxima, first quartile, median, third quartile, minima. Different letters above the bars indicated statistically significant differences between the samples (Brown-Forsythe ANOVA analysis followed by Dunnett's T3 multiple comparisons test, $p < 0.05$. Adjustments were made for multiple comparisons test).

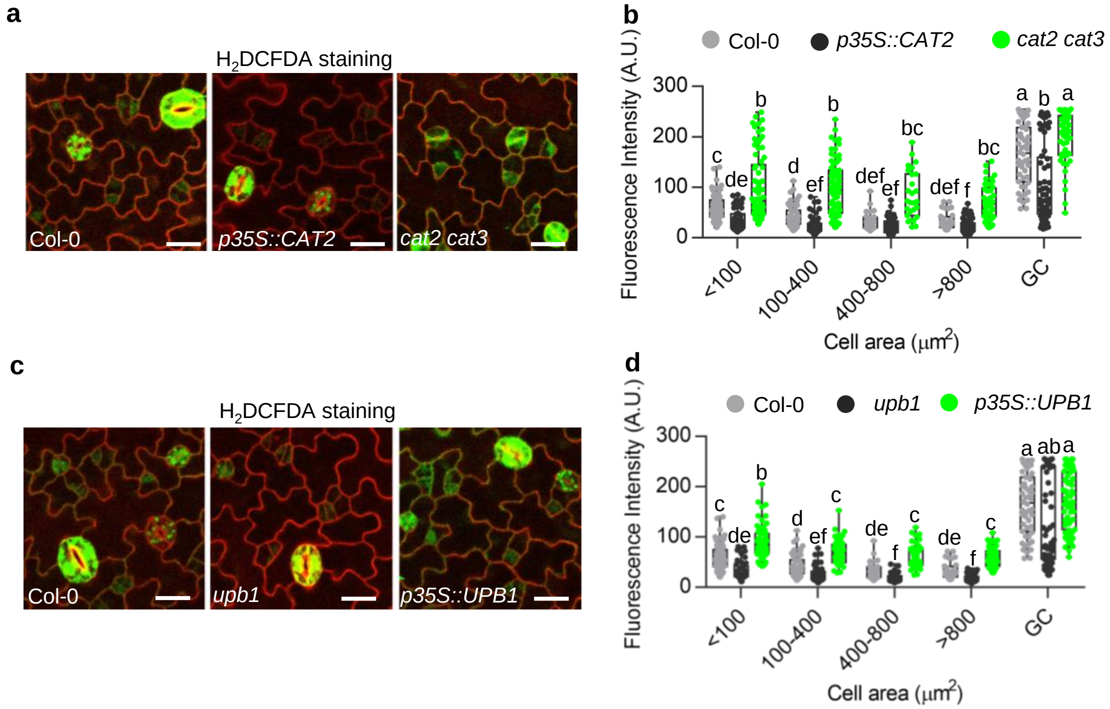

Supplementary Fig. 9 H_2DCFDA staining for H_2O_2 in the epidermal cells of wild type and indicated plants.

a-b, Quantification of H_2DCFDA fluorescent intensities in cotyledon epidermal cells of wild type, *p35S::CAT2-Myc* and *cat2 cat3* plants. **c-d**, Quantification of H_2DCFDA fluorescent intensities in cotyledon epidermal cells of wild type, *upb1* and *p35S::UPB1* plants. Seedlings of Col-0 and indicated plants were grown on ½ MS solid medium containing 1% sucrose under 16h light/8h dark photoperiod with 100 µMol/m^2/s for 4 days. Fluorescent signals were taken using LSM700 microscope from Zeiss. Scale bars in confocal images represent 20 µm. GFP signal from more than 200 epidermal cells in 10 cotyledons were analyzed ImageJ software in (**b**), (**d**) respectively. Box plot shows maxima, first quartile, median, third quartile, minima. Different letters above the bars indicated statistically significant differences between the samples (Brown-Forsythe ANOVA analysis followed by Dunnett's T3 multiple comparisons test, $p < 0.05$. Adjustments were made for multiple comparisons test).

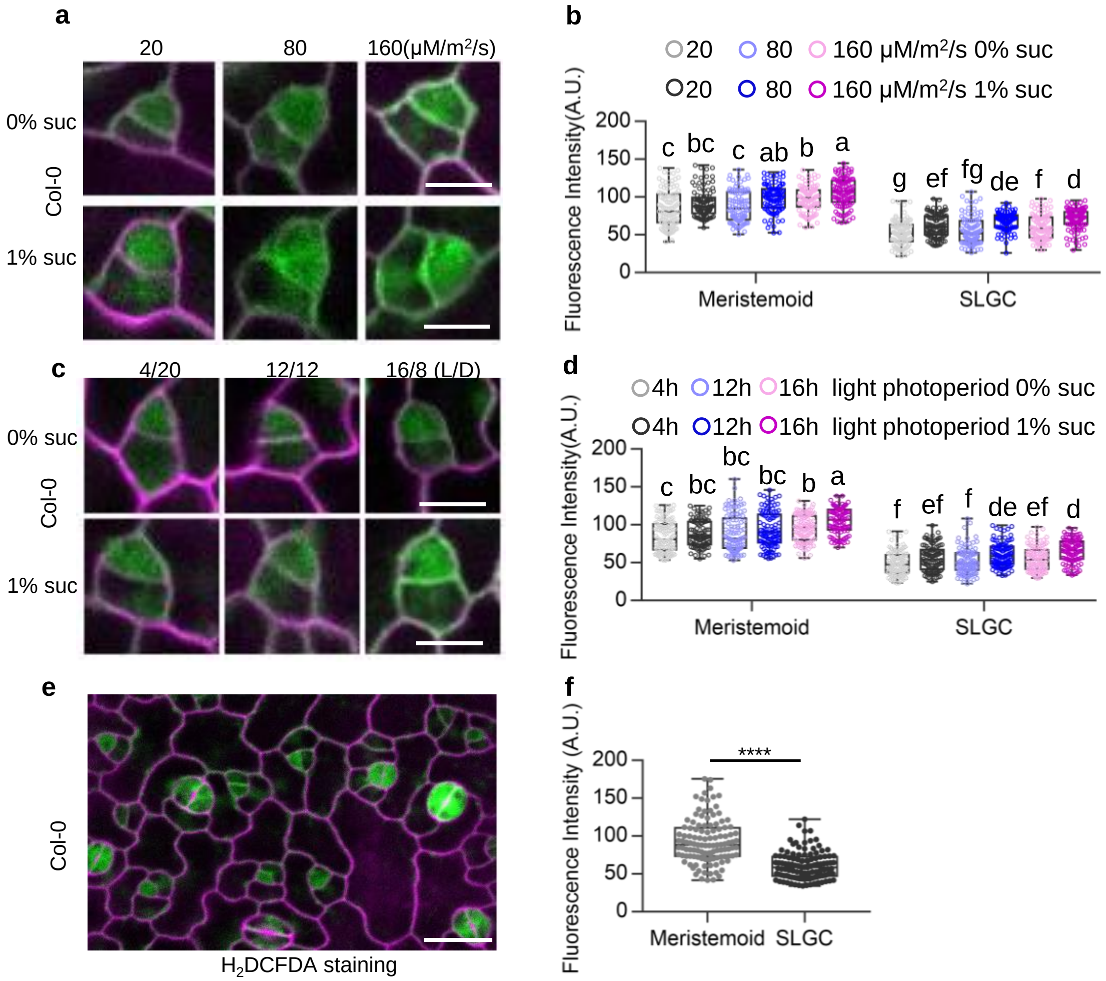

Supplementary Fig. 10 H_2O_2 specifically accumulates in the stomatal lineage cells under different growth conditions.

a-d, H_2DCFDA staining in the leaf epidermal cells of plants grown under different light intensities or different photoperiod conditions. n = 104, n = 105, n = 103, n = 102, n = 102, and n = 110 meristemoid or SLGC cells in 10 cotyledons were analyzed by ImageJ software in (**b**). n = 108, n = 106, n = 107, n = 105, n = 106, and n = 104 meristemoid or SLGC cells in 10 cotyledons were analyzed by ImageJ software in (**d**). Seedlings of Col-0 were grown on ½ MS solid medium with or without 1% sucrose under 16h light/8h dark photoperiod with different light intensities or under different photoperiod with 100 μMol/m^2/s for 4 days. **e-f**, H_2DCFDA staining in the true leaves. n = 116 meristemoid or SLGC cells in 10 true leaves were analyzed by ImageJ software in (**f**). Seedlings of Col-0 were grown on ½ MS solid medium containing 1% sucrose under 16h light/8h dark photoperiod with 100 μMol/m^2/s for 12 days.

Fluorescent signals were taken using LSM700 microscope from Zeiss. Scale bars in confocal images represent 10 μm in (**a**, **c**) and in (**e**) represent 20 μm. Box plot shows maxima, first quartile, median, third quartile, minima. Different letters above the bars indicated statistically significant differences between the samples (Brown-Forsythe ANOVA analysis followed by Dunnett's T3 multiple comparisons test, $p < 0.05$. Adjustments were made for multiple comparisons test). Asterisk between the bars indicated statistically significant differences between the samples (Two-tailed student's t test, ****$p < 0.0001$).

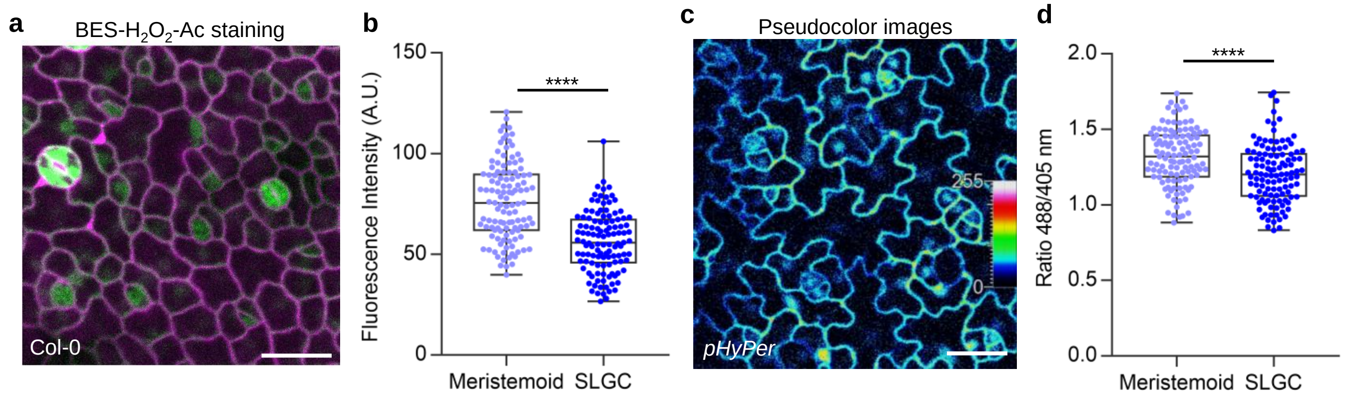

Supplementary Fig. 11 H_2O_2 specifically accumulates in the stomatal lineage cells of rosette leaves.

a-b, BES-H_2O_2-Ac staining for H_2O_2 in the epidermal cells of rosette leaves. The BES-H_2O_2-Ac fluorescent signals were in green, PI-marked cell outlines were in purple. Seedlings of Col-0 were grown on ½ MS solid medium containing 1% sucrose under 16h light/8h dark photoperiod with 100 µMol/m^2/s for 12 days. **c-d**, Quantification of *pHyPer* fluorescent signals in the epidermal cells of rosette leaves. Ratio imaging of Arabidopsis epidermal cells expressing *HyPer*. Seedlings of *pHyPer* transgenic plants were grown on ½ MS solid medium containing 1% sucrose under 16h light/8h dark photoperiod with 100 µMol/m^2/s for 12 days.

Fluorescent signals were taken using LSM700 microscope from Zeiss. n = 116 (BES-H_2O_2-Ac), n = 114 (*pHyPer*) meristemoid or SLGC cells in 10 true leaves were analyzed by ImageJ software in (**b**), (**d**) respectively. Scale bars in confocal images represent 20 µm. Box plot shows maxima, first quartile, median, third quartile, minima. Asterisk between the bars indicated statistically significant differences between the samples (Two-tailed student's t test, ****$p < 0.0001$).

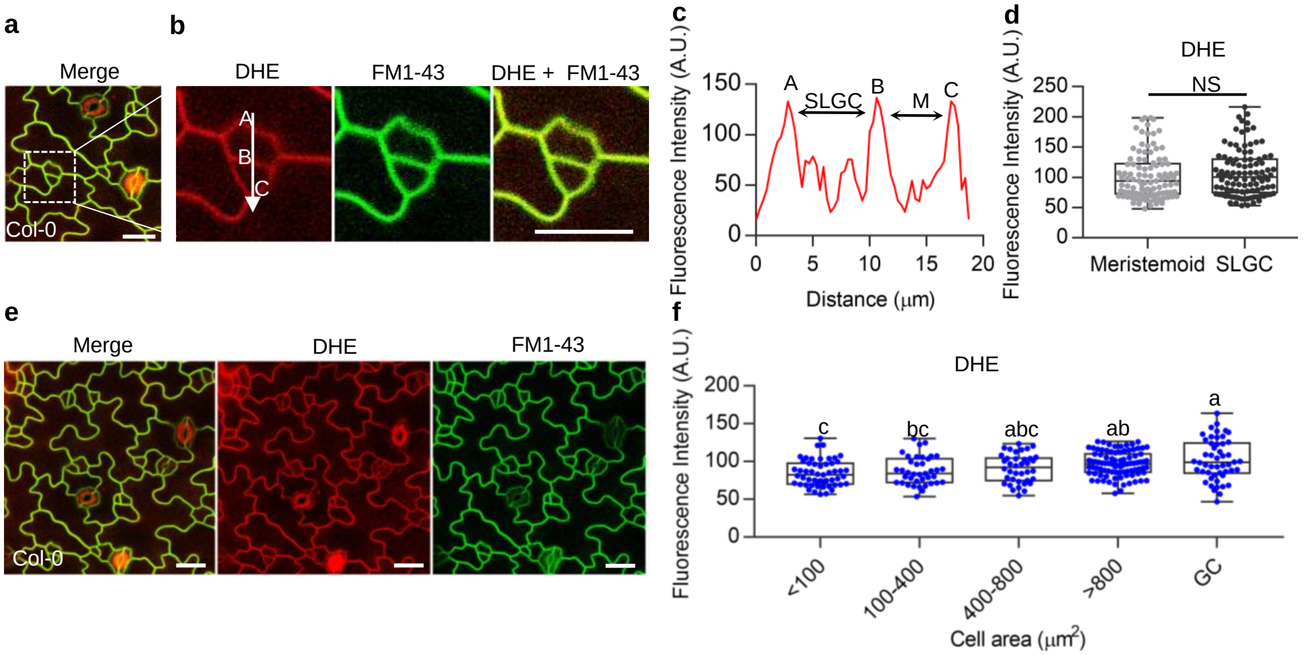

Supplementary Fig. 12 DHE staining for $O_2^{\cdot -}$ in the epidermis cell of leaves.

a-f, Measurement of $O_2^{\cdot -}$ in the epidermal cells of wild type cotyledon using DHE staining. Seedlings of Col-0 were grown on ½ MS solid medium containing 1% sucrose under 16h light/8h dark photoperiod with 100 μMol/m²/s for 4 days. Magnifications of the meristemoid and SLGC are shown on the (**b**). The fluorescent signals of DHE were determined along a line drawn on the confocal images using ImageJ software. n = 101 meristemoid or SLGC cells in 10 cotyledons were analyzed in (**d**). RFP signal from more than 200 epidermal cells in 10 cotyledons were analyzed in (**f**). The white arrows inside the images show the areas used for line scan measurements that yielded plot profiles shown in the right panels. The arrow labeling with A and B represents the fluorescent signals in SLGC, and the arrow labeling with B and C represents the fluorescent signals in meristemoid cells. Box plot shows maxima, first quartile, median, third quartile, minima. Different letters above the bars indicated statistically significant differences between the samples (Brown-Forsythe ANOVA analysis followed by Dunnett's T3 multiple comparisons test, $p < 0.05$. Adjustments were made for multiple comparisons test). Scale bars in confocal images represent 20 μm.

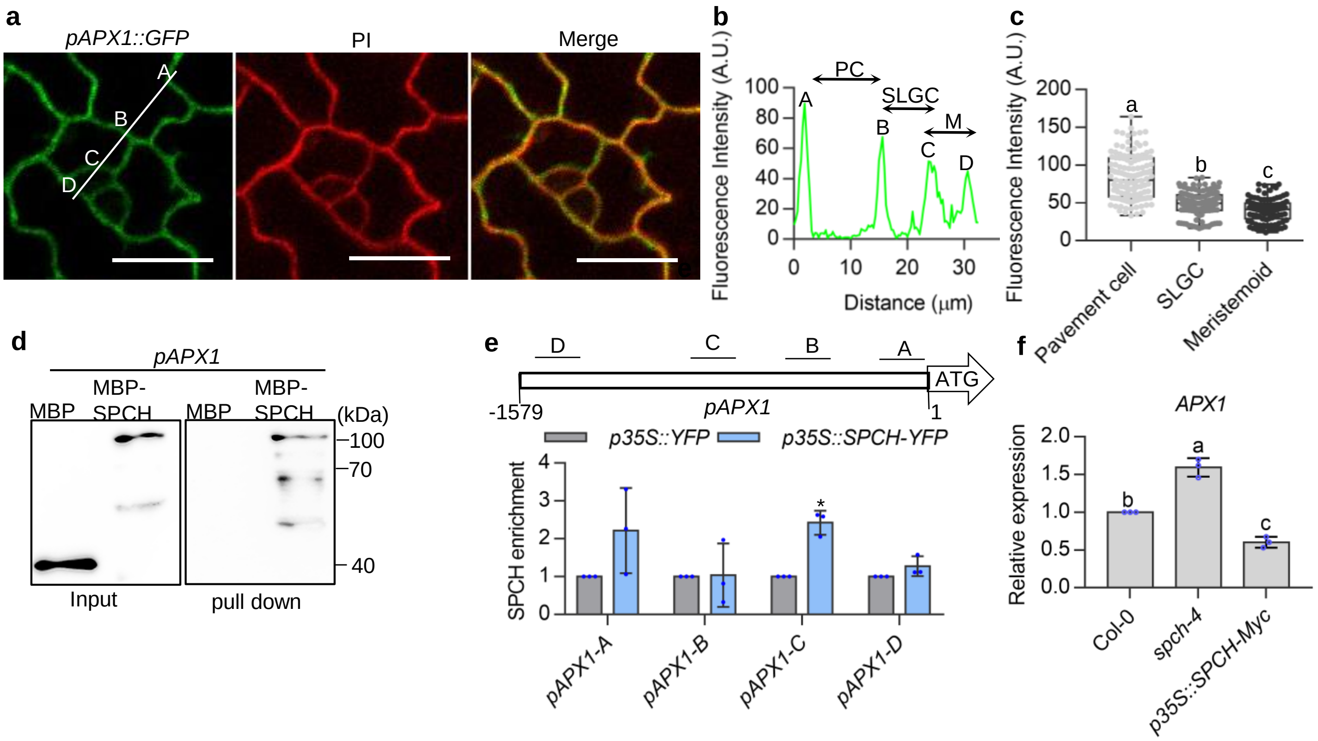

Supplementary Fig. 13 ***APX1*** **highly expresses in the pavement cells of leaves.**

a-c, The expression pattern of *APX1* in the cotyledon epidermal cells. Seedlings of *pAPX1::GFP* transgenic plants were grown on ½ MS solid medium containing 1% sucrose under 16h light/8h dark photoperiod with 100 μMol/m^2/s for 4 days. The GFP fluorescent signals were in green, PI-marked cells for outlines of cell were in red. Mergence images showed the higher *APX1* expression levels in the pavement cells and the lower levels in the meristemoid cells. Box plot shows the positive relationship between the *pAPX1::GFP* fluorescent intensities and epidermal cell area in the cotyledon. n = 127 (Pavement), n = 110 (SLGC) and n = 110 (meristemoid) cells from 10 cotyledons were examined in (**c**). Box plot shows maxima, first quartile, median, third quartile, minima. Different letters above the bars indicated statistically significant differences between the samples (Brown-Forsythe ANOVA analysis followed by Dunnett's T3 multiple comparisons test, $p < 0.05$. Adjustments were made for multiple comparisons test). Scale bars in confocal images represent 20 μm. **d**, SPCH directly binds to the promoter of *APX1 in vitro*. MBP or MBP-SPCH were incubated with biotinylated DNA fragments from the *APX1* promoter immobilized on streptavidin beads. The DNA-bound proteins were immunoblotted using anti-MBP antibody. **e**, Quantitative ChIP-PCR showed that *APX1* is the target gene of SPCH. Seedlings of *p35S::YFP* and *p35S::SPCH-YFP* were used to performed ChIP assays. The levels of SPCH binding were calculated as the ratio between *p35S::SPCH-YFP* and *p35S::YFP*, and then normalized to that of control gene *PP2A*. Error bars indicate standard deviation (S.D.). $^*P < 0.05$, as determined by a Two-tailed student`s t-test (n=3 biologically independent samples). **f**, RT-qPCR analysis of the expression of *APX1* in wild type, *spch-4* mutant and *p35S::SPCH-Myc* transgenic plants.. Seedlings were grown on ½ MS solid medium containing 1% sucrose under 16h light/8h dark photoperiod with 100 μMol/m^2/s for 6 days. *PP2A* gene was used as an internal control. Error bars indicate standard deviation (S.D) (n=3 biologically independent samples). Different letters above the bars indicated statistically significant differences between the samples (One-way ANOVA analysis followed by Tukey's multiple comparisons test, $p < 0.05$. Adjustments were made for multiple comparisons test).

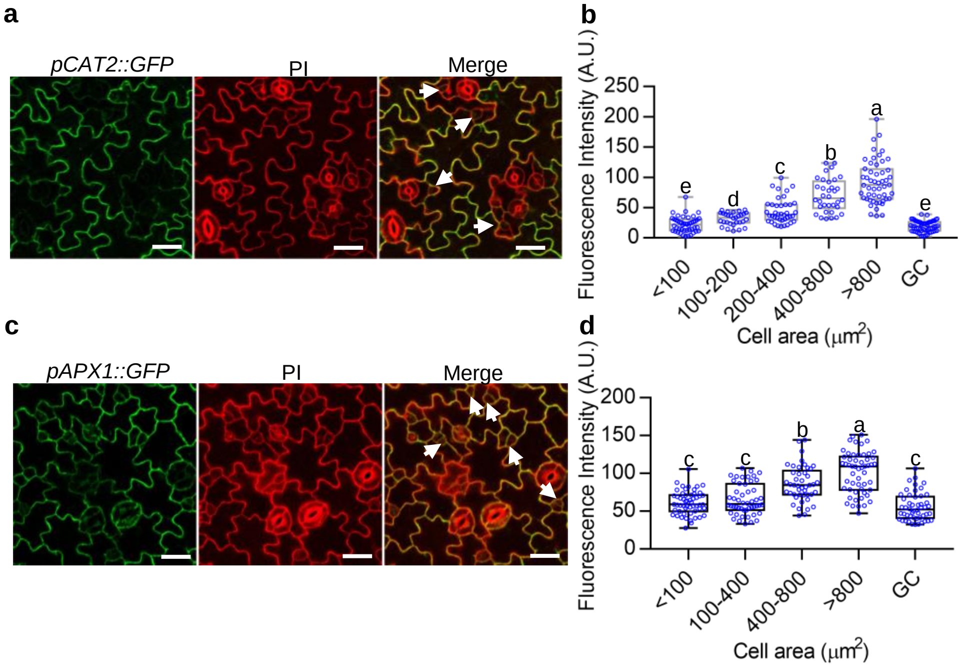

Supplementary Fig. 14 The expression patterns of *CAT2* and *APX1* in the epidermal cells of leaves.

a-b, The expression pattern of *CAT2* in the cotyledon epidermal cells. **c-d**, The expression pattern of *APX1* in the cotyledon epidermal cells. Seedlings of *pCAT2::GFP* and *pAPX1::GFP* were grown on ½ MS solid medium containing 1% sucrose under 16h light/8h dark photoperiod with 100 μMol/m^2/s for 4 days. The GFP fluorescent signals were in green, PI-marked cells for outlines of cell were in red. Merged images showed the higher expression levels of *CAT2* or *APX1* in the pavement cells and the lower levels in the smaller cells where cell division occurs. Box plot shows the positive relationship between the fluorescent intensities of *pCAT2::GFP* or *pAPX1::GFP* and epidermal cell area in the cotyledon. GFP signal from more than 200 epidermal cells in 10 cotyledons were analyzed by ImageJ software in (**b**), (**d**) respectively. Scale bars in confocal images represent 20 μm. Box plot shows maxima, first quartile, median, third quartile, minima. Different letters above the bars indicated statistically significant differences between the samples (Brown-Forsythe ANOVA analysis followed by Dunnett's T3 multiple comparisons test, $p < 0.05$. Adjustments were made for multiple comparisons test).

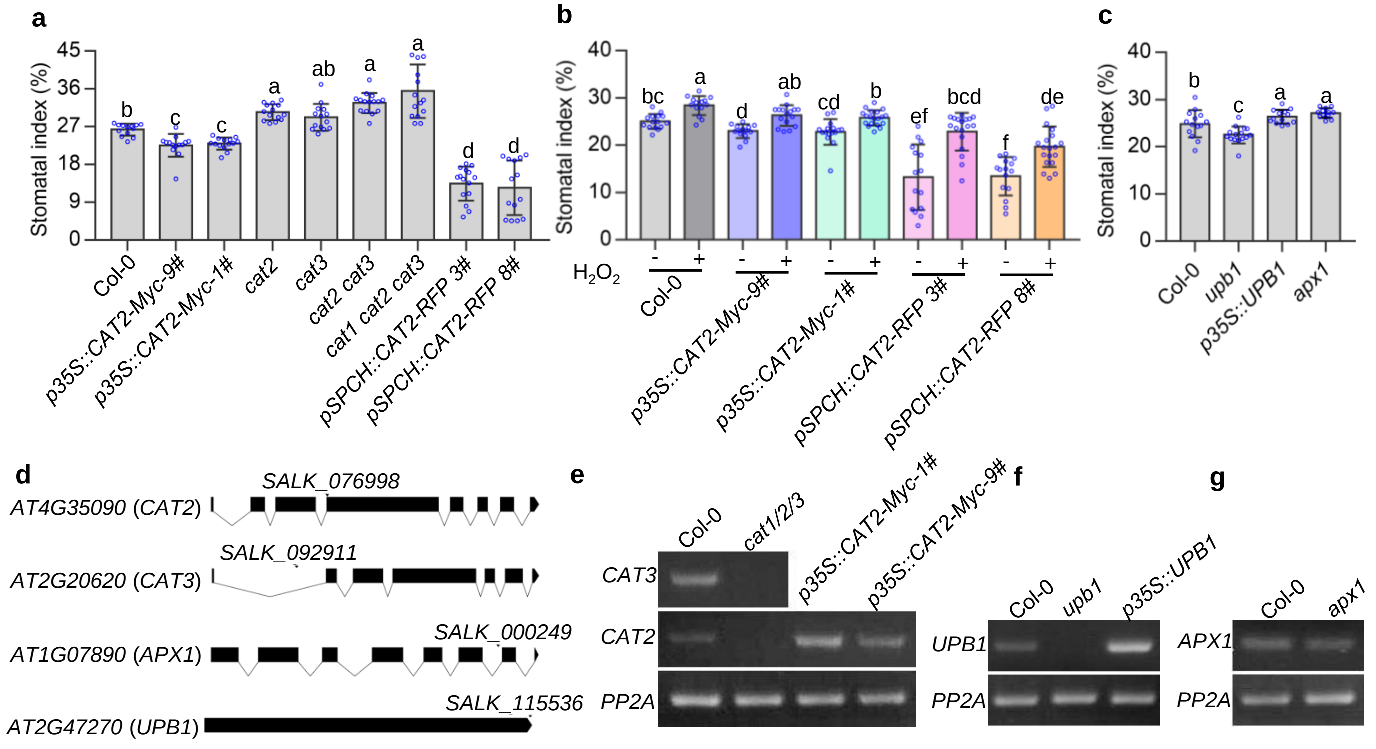

Supplementary Fig. 15 Ectopic expression of *CAT2* results in the decreased stomatal index.

a-c, Quantification of stomatal index of wild type and indicated plants. *n* = 13, 14, 14, 14, 14, 15, 15, 15, 14 independent cotyledons were examined in (**a**). *n* = 15, 14, 14, 16, 16, 19, 15, 19, 15, 18 independent cotyledons were examined in (**b**). *n* = 14, 15, 13, 12 independent cotyledons were examined in (**c**). Seedlings of wild type and diverse mutants were grown on ½ MS solid medium containing 1% sucrose with or without 10 μM H_2O_2 for 8 days under 16h light/8h dark photoperiod with 100 μMol/m^2/s, Error bars indicate standard deviation (S.D.). Different letters above the bars indicated statistically significant differences between the samples (Brown-Forsythe ANOVA analysis followed by Dunnett's T3 multiple comparisons test, $p < 0.05$ (**a**, **b**). Adjustments were made for multiple comparisons test; One-way ANOVA analysis followed by Uncorrected Fisher's LSD multiple comparisons test, $p < 0.05$ (**c**). No adjustments were made for multiple comparisons test). **d**, Schematic diagram showing the T-DNA insertion sites in the indicated mutants. **e-g**, RT-PCR analysis of the expression of indicated genes in Col-0 and different mutans. *PP2A* was used as the internal control.

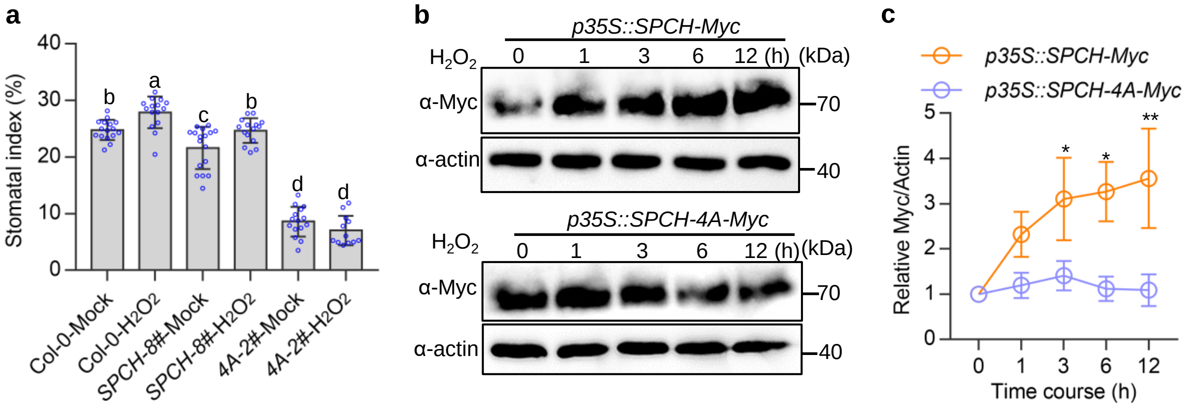

Supplementary Fig. 16 H_2O_2 fails to promote stomatal development on the *pSPCH::SPCH-4A-Myc/spch-4* transgenic plants.

a, Quantification of stomatal index of wild type and indicated plants. *n* = 16, 15, 17, 15, 15, 12 independent cotyledons were examined in (**a**). Seedlings of Col-0 and *pSPCH::SPCH-RFP/spch-4* (*SPCH-8#*) and *pSPCH::SPCH-4A-RFP/spch-4* (*4A-2#*) were grown on ½ MS solid medium containing 1% sucrose with or without 10 μM H_2O_2 under 16 h light/8h dark photoperiod with 100 μMol/m^2/s for 8 days, Different letters above the bars indicated statistically significant differences between the samples (One-way ANOVA analysis followed by Tukey's multiple comparisons test, $p < 0.05$. Adjustments were made for multiple comparisons test). Error bars indicate standard deviation (S.D.). **b-c**, Quantification of the effects of H_2O_2 on SPCH or SPCH-4A protein. Seedlings of *p35S::SPCH-Myc* and *p35S::SPCH-4A-Myc* transgenic plants were grown on ½ MS solid medium containing 1% sucrose under 16 h light/8h dark photoperiod with 100 μMol/m^2/s for 7 days, then treated with 1 mM H_2O_2 for different times. Error bars indicate standard deviation (S.D.). (n=3 biologically independent samples). Asterisk between the bars indicated statistically significant differences between the samples (Two-tailed student's t test, $^{**}p < 0.01$, $^{*}p < 0.05$).

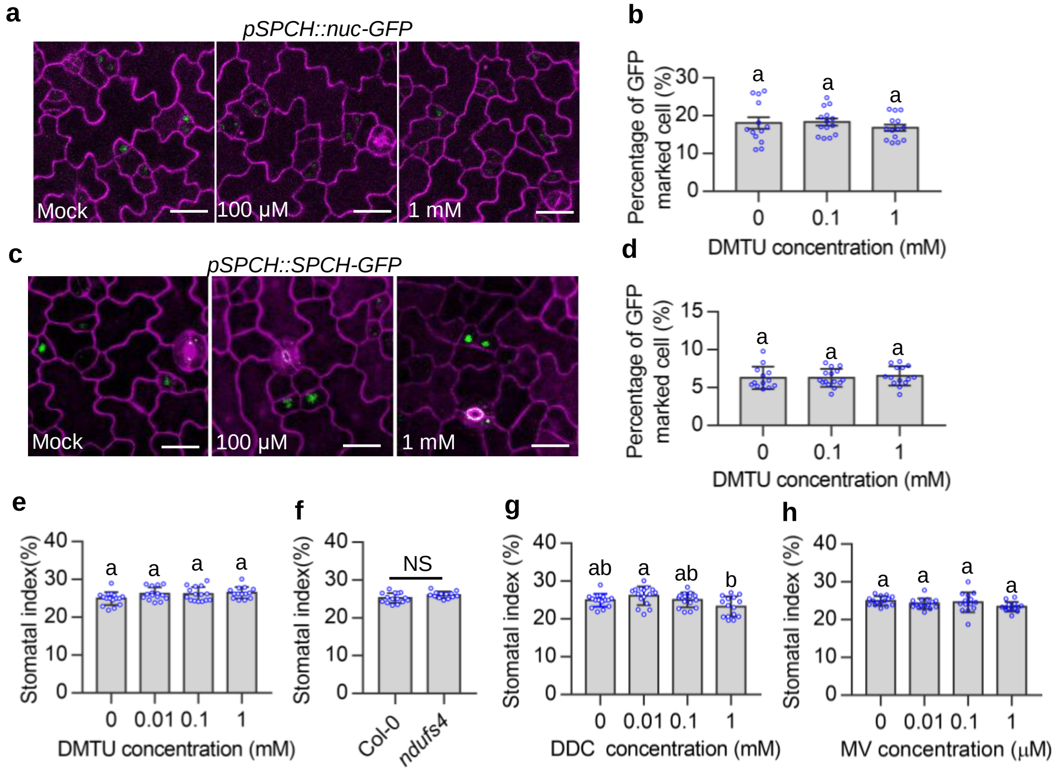

Supplementary Fig. 17 $O_2^{\bullet-}$ has no marked effects on the stomatal development.

a-d, DMTU had no significant effects on cell fate of the epidermal cells in Arabidopsis leaves. *n* = 13, 14, 14 independent cotyledons were examined in (**b**). *n* = 13, 15, 14 independent cotyledons were examined in (**d**). Seedlings of *pSPCH::nucGFP*, and *pSPCH::SPCH-GFP* were grown on ½ MS solid medium containing 1% sucrose under 16h light/8h dark photoperiod with 100 μMol/m^2/s in the presence of different concentrations of DMTU for 3 days. Scale bars in confocal images represent 20 μm. **e**, Quantification of the effects of DMTU on stomatal index. *n* = 16, 15, 15, 14 independent cotyledons were examined in (**e**). **f**, Quantification of the stomatal index of wild type plants and *ndufs4* mutant. *n* = 15, 13 independent cotyledons were examined in (**f**). Seedlings of Col-0 and *ndufs4* were grown in ½ MS solid medium containing 1% sucrose under 16h light/8h dark photoperiod with 100 μMol/m^2/s for 8 days. ns represents the no significant difference between Col-0 and *ndufs4* mutants. **g**,**h**, Quantification of the effects of DDC and MV on stomatal index. *n* = 16, 16, 15, 15 independent cotyledons were examined in (**g**). *n* = 15, 15, 14, 14 independent cotyledons were examined in (**h**).

Seedlings of Col-0 were grown in ½ MS solid medium containing 1% sucrose in the presence of different concentrations of DMTU, DDC or MV under 16h light/8h dark photoperiod with 100 μMol/m^2/s for 8 days. Different letters above the bars indicated statistically significant differences between the samples (One-way ANOVA analysis followed by Tukey's multiple comparisons test, $p < 0.05$. Adjustments were made for multiple comparisons test). Error bars indicate standard deviation (S.D.).

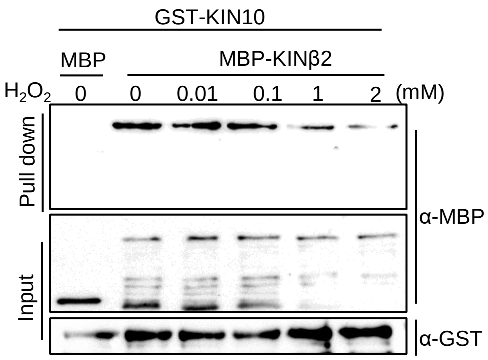

Supplementary Fig. 18 H_2O_2 reduces the interaction between KIN10 and KINβ2 in a dose-dependent manner.

H_2O_2 reduces the interaction between KIN10 and KINβ2 in vitro. MBP and MBP-KINβ2 were incubated with GST-KIN10 bound to glutathione agarose beads with or without different concentration H_2O_2 for 1 h, and then eluted and analyzed by immunoblotting using anti-MBP antibody.

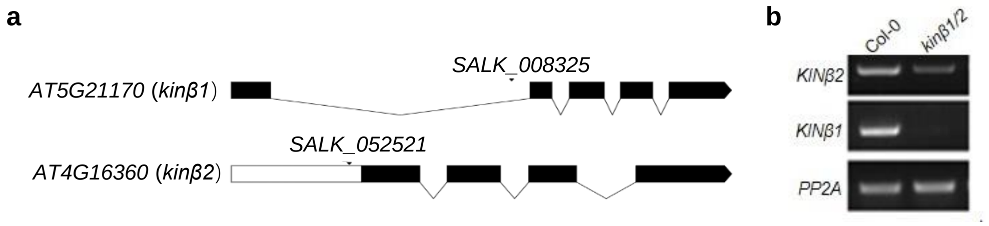

Supplementary Fig. 19 The information of *KINβ1* and *KINβ2* T-DNA mutants.

a, Schematic diagram showing the T-DNA insertion sites in the genomic region *KINβ1* and *KINβ2*, respectively. **b**, RT-PCR analysis of the expression of *KINβ1* and *KINβ2* in Col-0 and *kinβ1 kinβ2* mutants. Seedlings of Col-0 and *kinβ1 kinβ2* mutants were grown in ½ MS solid medium containing 1% sucrose under 16h light/8h dark photoperiod with 100 μMol/m^2/s for 7 days. *PP2A* was used as the internal control.

Fig. 3i

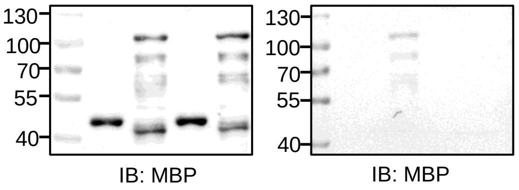

Fig. 5a

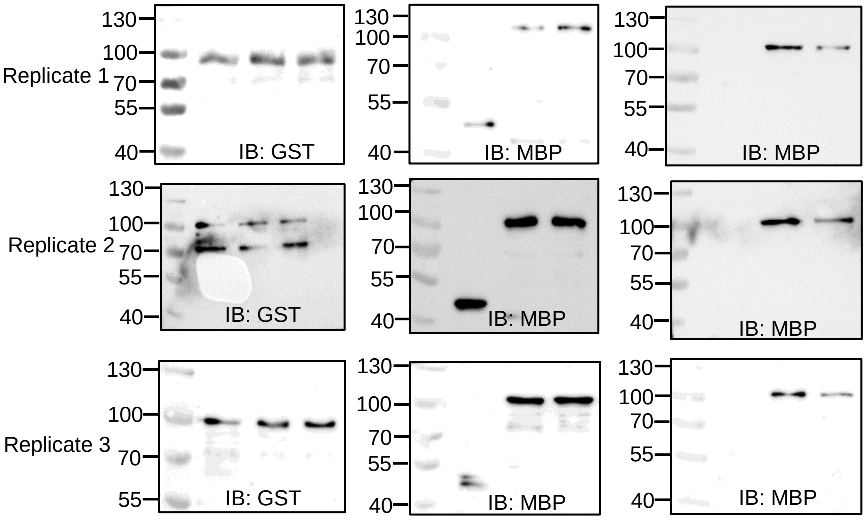

Fig. s13d

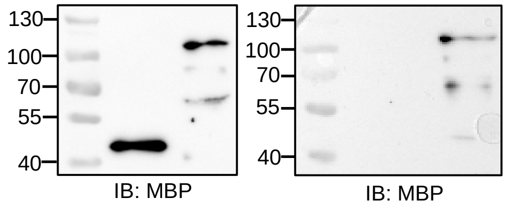

Fig. 5c

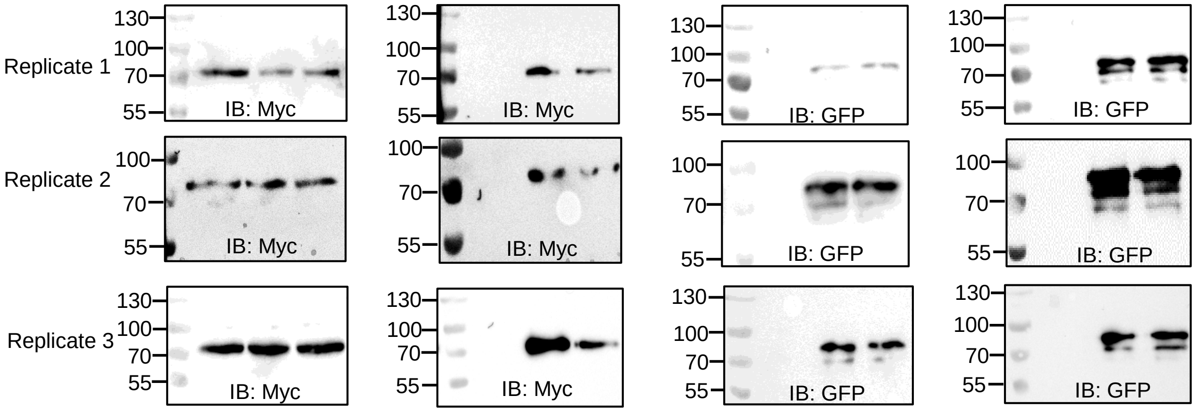

Fig. s16b

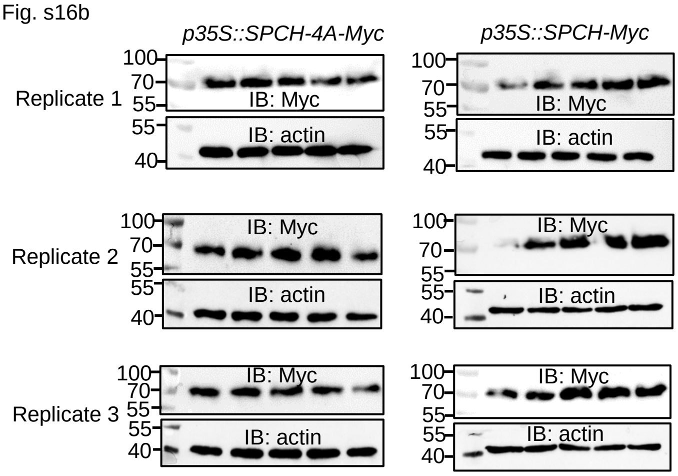

Fig. s18

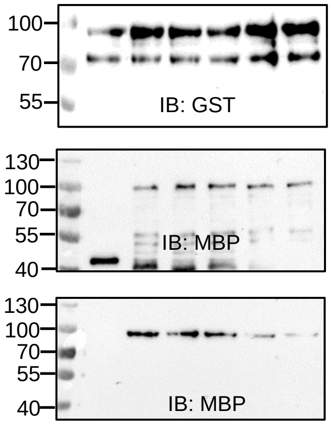

Supplementary Fig. 20 Full scan data of original immunoblots.
