# Supplementary material for: Spatially patterned hydrogen peroxide orchestrates stomatal development in Arabidopsis
Source: Nat Commun. 2022 Aug 26;13:5040. doi: 10.1038/s41467-022-32770-7 (PMC9418256; doi:10.1038/s41467-022-32770-7)
Supplement: Supplementary file 3 — Description of Additional Supplementary Files [file 41467_2022_32770_MOESM3_ESM.pdf]

Description of Additional Supplementary Files

File Name: Supplementary Data 1
Description: Oligonucleotide sequences used in this paper.
